# Supplementary material for: Bayesian refinement of protein structures and ensembles against SAXS data using molecular dynamics
Source: PLoS Comput Biol. 2017 Oct 18;13(10):e1005800. doi: 10.1371/journal.pcbi.1005800 (PMC5662244; doi:10.1371/journal.pcbi.1005800)
Supplement: S1 Archive — (BZ2) [file pcbi.1005800.s011.tar.bz2 › gromacs_4.62_bayesian_saxs_refi/share/top/amber99sb-star-ildn.ff/forcefield.doc]

AMBER99SB* modification of ff99SB (Best and Hummer, J. Phys. Chem. B, 113, 9004-9015, 2009)

combined with

AMBER99SB-ILDN force field (Lindorff-Larsen et al., Proteins 78, 1950-58, 2010)

********************************************************************

* Amber99sb-ildn is an improvement to Amber99SB by	*

* Kresten Lindorff-Larsen and coworkers at D.E. Shaw Research, inc.*

* The primary publication (please cite it) is	*

* Lindorff-Larsen, K., Piana, S., Palmo, K., Maragakis, P., *

* Klepeis, J.L., Dror, R.O., Shaw, D.E., *

* Improved side-chain torsion potentials for the Amber ff99SB	*

* protein force field, Proteins 78:1950–1958 (2010)	*

********************************************************************
